# Mortality, cardiovascular disease, and cancer in coeliac disease and dermatitis herpetiformis: a matched cohort study

**DOI:** 10.1016/j.lana.2026.101512

**Published:** 2026-05-28

**Authors:** Jonas F. Ludvigsson, Jason Dagher, Benjamin Lebwohl, Jialu Yao, Peter H.R. Green, Ralf J. Ludwig, Philip Curman

**Affiliations:** a Department of Medical Epidemiology and Biostatistics, Karolinska Institutet, Stockholm, Sweden; b Department of Pediatrics, Örebro University Hospital, Sweden; c Celiac Disease Center, College of Physicians and Surgeons, Columbia University Irving Medical Center, New York, NY, USA; d Department of Dermatology, Université de Sherbrooke, Quebec, Canada; e Department of Epidemiology, Columbia University, New York, USA; f Institute and Comprehensive Center for Inflammation Medicine, University-Hospital Schleswig-Holstein (UKSH), Lübeck, Germany; g Lübeck Institute of Experimental Dermatology, University of Lübeck, Lübeck, Germany; h Dermato-Venereology Clinic, Karolinska University Hospital, Stockholm, Sweden; i Division of Dermatology and Venereology, Department of Medicine (Solna), Karolinska Institutet, Stockholm, Sweden

**Keywords:** Coeliac disease, Dermatitis herpetiformis, Cancer, Death, Major adverse cardiovascular events

## Abstract

**Background:**

Recent data on mortality risk in coeliac disease (CeD) have been contradictory. Large-scale research on mortality, cardiovascular disease (CVD), and cancer in dermatitis herpetiformis (DH) remains scarce.

**Methods:**

Using the U.S. TriNetX database we identified 204,056 adults with CeD and 6896 with DH between 2005 and 2025, and individually matched 1:1 with comparators without CeD or DH. Cox regression estimated hazard ratios (HRs) for mortality, CVD, and cancer, matching cohorts based on age, sex, nicotine dependence, and comorbidities.

**Findings:**

The mean age of patients with CeD and DH was 42.7 and 53.1 years, respectively, and females constituted 70.5% and 53.2%, respectively. Among patients with CeD, 8791/200551 (4.38%) died, compared with 7841/201285 (3.90%) matched comparators (adjusted (a)HR = 1.18; 95% CI = 1.14–1.22). Patients with CeD showed a modestly increased risk of a major adverse cardiovascular event (MACE, aHR = 1.11; 1.08–1.14). CeD was associated with an increased risk of haematological cancers (aHR = 1.42; 1.32–1.53), including non-Hodgkin lymphoma (aHR = 1.51; 1.35–1.68), but not with solid cancers (aHR = 1.00; 0.97–1.03). Among patients with DH, 566/6768 (8.36%) died, compared with 443/6770 (6.54%) matched comparators (aHR = 1.25; 1.10–1.42). DH was linked to MACE (aHR = 1.22; 1.08–1.37) and non-Hodgkin lymphoma (aHR = 2.58; 1.47–4.51).

**Interpretation:**

This U.S. population-based cohort study found that patients with CeD or DH had increased mortality risks compared with matched comparators. Both patient groups were also at increased risk of CVD, including MACE. Cancer associations showed different patterns in CeD and DH, with both exhibiting increased risks of non-Hodgkin lymphoma, with the highest risk observed in DH.

**Funding:**

Region Stockholm and Karolinska Institutet.

**Research in context**

**Evidence before this study**

We searched PubMed from database inception to May 2025 for population-based studies, cohort studies, systematic reviews, and meta-analyses using the terms “coeliac disease” OR “celiac disease” OR “dermatitis herpetiformis” combined with “mortality”, “death”, “cardiovascular disease”, “major adverse cardiovascular events”, “myocardial infarction”, “stroke”, “heart failure”, “arrhythmia”, “cancer”, “malignancy”, “lymphoma”, “non-Hodgkin lymphoma”, and “gastrointestinal cancer”. We also reviewed the reference lists of relevant articles. The available evidence on mortality, cardiovascular disease, and cancer in coeliac disease was inconsistent, especially for mortality and cardiovascular disease, and was derived largely from European cohorts. For dermatitis herpetiformis, large-scale studies of these outcomes were limited, with sparse data on cardiovascular disease and cancer and little evidence from non-European populations.

**Added value of this study**

This study extends existing evidence by providing large-scale data from a U.S. population in a field where most prior evidence has come from Europe and where findings, especially for mortality and cardiovascular disease in coeliac disease (CeD), have been inconsistent. It also addresses a major knowledge gap for DH, for which large-scale data on mortality, cardiovascular disease, and cancer remain limited. By evaluating both conditions across multiple clinically relevant outcomes in a uniform framework, the study helps define which adverse outcomes appear to be shared across the CeD spectrum and which may differ between intestinal and cutaneous disease manifestations.

**Implications of all the available evidence**

Our findings will underscore the need for multidisciplinary efforts to prevent complications and adverse outcomes in both disorders. Indeed, this study highlights the need for emphasis on cardiovascular and cancer risk factors in clinical management of patients with CeD and DH. The observed excess mortality and differing cancer risks suggest distinct underlying immunoinflammatory mechanisms. Understanding these pathways may reveal why related autoimmune diseases follow divergent clinical trajectories. Further research is warranted to elucidate the biological mechanisms driving excess mortality and variable cancer risks in CeD and DH.

## Introduction

Coeliac disease (CeD) is an immune-mediated disorder triggered by gluten exposure and occurring in genetically sensitive individuals.^1^ While the largest study to date has demonstrated an excess mortality in patients with biopsy-confirmed CeD (hazard ratio, HR 1.14; 1.10–1.18),^2^ one British study using primary care data,^3^ and one Finnish study based on insurance data on CeD found no increased mortality.^4^ Furthermore, a recent meta-analysis reported a weighted HR of 1.29 for death in diagnosed CeD.^5^ High heterogeneity (I^2^ = 93%) was reported, underlining the variability in risk estimates, while also noting an absence of population-based CeD-mortality studies outside of Europe.

The literature on CeD and cardiovascular disease (CVD) presents inconsistent findings. Studies from the UK and Sweden respectively reported a +27% increased risk of CVD in patients with CeD^6^ and a 19% increased risk of ischemic heart disease,^7^ both of which were statistically significant. While one meta-analysis demonstrated an increased risk of stroke (HR 1.10; 1.01–1.19), risk estimates for myocardial infarction (HR 1.12) and cardiovascular death (HR 1.12) failed to attain statistical significance.^8^ These findings aligned with a recent meta-analysis, which indicated an HR of 1.14 (95% CI 0.62–2.11) for myocardial infarction in CeD.^9^ Hence, a positive association between CeD and CVD is probable, but existing studies may have been underpowered to detect a minor risk increase.^8^^,^^9^

Evidence on cancer in CeD is more extensive. In our 2022 publication, we examined about 47,000 individuals with CeD in Sweden and found a minimally increased risk of any cancer (HR 1.11; 1.07–1.15).^10^ Lymphoproliferative and gastrointestinal (GI) cancers had the highest relative risk estimates, while an inverse relationship was observed for breast cancer. This is in line with another study which reported an increased risk of death from lymphoproliferative disease,^4^ but a non-significant association with death from GI cancer. Finally, a recent French study reported increased risks of non-Hodgkin lymphoma as well as for most GI cancers, but a decreased risk of breast cancer in patients with CeD.^11^ Although the literature supports a positive cancer association, selection bias in some studies may have led to risk overestimation. Furthermore, existing evidence, with minor exceptions,^12^ is largely limited to Europe.^13^ This supports the need for studies analysing these associations in other populations, notably on the American continent.

Another population of interest is patients presenting with dermatitis herpetiformis (DH), a cutaneous manifestation of CeD. It is characterised by intensely pruritic vesicles and papules, typically distributed symmetrically on extensor surfaces, and diagnosis is confirmed through direct immunofluorescence. The highest incidence is in Northern European regions, with a prevalence of 1.2–39.2 per 100,000 individuals.^14^ In Finland and the UK, where adherence to a gluten-free diet (GFD) is generally high,^15^ several large cohort studies on DH have reported no increase in all-cause mortality.^16^, ^17^, ^18^, ^19^ Two studies even revealed a statistically significant reduction in standardised mortality ratio (SMR) compared to the general population (0.52^20^ and 0.72^21^). While another study with a 30-year follow-up also found a SMR of 0.72 for ischemic heart disease, 0.46 for cerebrovascular disease, and 0.72 for circulatory system disease.^20^

On the other hand, cardiovascular and cancer outcomes in DH have received limited investigation. A recent Finnish study, involving 368 biopsy-confirmed patients with DH and 1099 matched controls, found no increased hazard for CVD after adjusting for diabetes at baseline.^22^ Similarly, a Turkish study found no risk excess for CVD in DH, although the sample size was small.^23^ Most studies on cancer in DH have reported risk elevations for lymphoproliferative malignancies, whereas the overall cancer risk may not be increased.^24^ In particular, a UK study found no association of DH with either overall or lymphoproliferative malignancies,^19^ while other studies indicate a many-fold increased lymphoma risk, primarily within the first five years post-diagnosis and in patients not adhering to a GFD.^17^^,^^25^ This highlights a gap in large-scale studies focused on assessing CVD and cancer risk in patients with DH to support clinical prevention strategies.

In summary, earlier data on mortality in CeD are inconsistent, and large-scale studies on DH mortality are lacking. Moreover, there is a scarcity of population-based studies on CeD and DH in non-European countries. We therefore examined mortality, CVD, and cancer risk in more than 200,000 patients with CeD and almost 7000 with DH in a U.S. population-based setting.

## Methods

### Database

This investigation used electronic health records from the TriNetX database, which provides secure access to detailed healthcare data.^26^^,^^27^ Data from over 129 million patients across 71 healthcare organisations in the United States Collaborative Network were analysed. The study period started in May 2005, with variable individual entry points, and ended in May 2025. The TriNetX data include aggregated information on demographics, diagnoses (coded using the *International Classification of Diseases, 10th edition, Clinical Modification* [ICD-10-CM]), medical procedures, prescribed medications, laboratory tests, and records of healthcare use (see Appendix for details on data sources).

### Study design and population

**Fig. 1:**
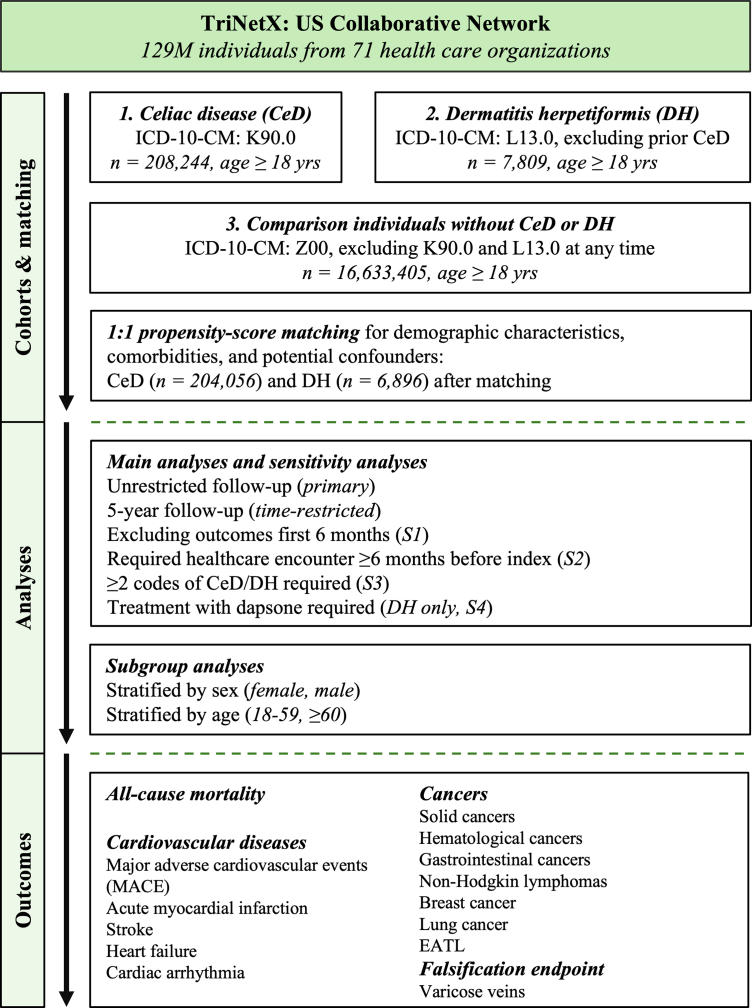
Flowchart of the study design. Cohort numbers in the upper boxes represent those before propensity-score matching; cohort numbers after matching appear below. CeD: coeliac disease; DH: dermatitis herpetiformis; EATL: enteropathy-associated T-cell lymphoma.

A large-scale, retrospective, propensity-score matched (PSM) cohort study was performed according to the STROBE guidelines^28^ and methodologically based on published investigations.^29^^,^^30^ Two primary cohorts were defined: (1) individuals with an incident diagnosis of CeD (ICD-10-CM: K90.0) and (2) individuals with an incident diagnosis of DH (ICD-10-CM: L13.0, excluding any instance of CeD before the first instance of DH). A comparison cohort comprising individuals without either CeD or DH was defined by an instance of a general healthcare encounter (ICD-10-CM: Z00). The date of earliest qualifying diagnosis or encounter was defined as the index event. Only individuals aged ≥ 18 years at the index event were eligible for inclusion. The study design is summarised in Fig. 1.

### Covariates and analytical framework

The selection of covariates for PSM was predetermined, considering their clinical significance and potential confounding effects. Matching variables included: age at diagnosis, female sex, nicotine dependence (ICD-10-CM:F17), essential hypertension (I10), and diabetes (E08-E13). Inclusion of additional covariates risked introducing model instability, reduced matching efficiency, and increased risk of incorporating variables that do not represent true confounders. Thus, we limited our PSM to clinically relevant confounders.

CeD and DH were analysed in parallel, using identical analytical frameworks. The primary analysis investigated outcomes from day one to any time after the index event, with an additional time-restricted analysis limiting follow-up to five years. Three main sensitivity analyses were performed: (1) exclusion of outcomes recorded within the first six months post-index to reduce detection bias and reverse causation; (2) implementing a required healthcare encounter ≥6 months before the index date to ensure adequate observation time and further mitigate detection bias; and (3) use of stricter cohort definitions requiring ≥2 diagnostic codes for CeD or DH to increase diagnostic validity. To further strengthen cohort validity, in a fourth sensitivity analysis limited to DH, we required both a DH diagnosis and documented dapsone treatment when identifying the cohort. Follow-up started at the later date between a DH diagnosis and the first dapsone record. We also performed two minor sensitivity analyses, including (i) matching for race/ethnicity (White, Black/African American, and Hispanic/Latino) apart from age and sex, and (ii) excluding any instance of IBD or GI cancer until time of CeD or DH diagnosis.

Varicose veins (ICD-10-CM:I83), a common and unrelated condition with no known epidemiologic association with either CeD or DH, was used as a falsification endpoint to assess residual confounding and surveillance bias. We expected non-significant associations between this condition and CeD or DH.

Additionally, subgroup analyses for the primary cohort definitions stratified by sex (male, female) and age at index (18–59, ≥60 years) were conducted. All sensitivity and subgroup analyses used an unrestricted follow-up.

### Outcomes

ICD-10-CM codes defined all outcomes. Outcomes included all-cause mortality (a record of “deceased status” in the database or R99); CVD-related outcomes were major adverse cardiovascular events (MACE; I21, I63, or I50), myocardial infarction (MI, I21), stroke (I63), heart failure (I50), and cardiac arrhythmia (I47–I49); in addition, we investigated different cancers: solid cancers (C00–C75), haematological cancers (C81–C96), GI cancers (C15–C20), non-Hodgkin lymphomas (C82–C86), breast cancer (C50), lung cancer (C34), and enteropathy-associated (intestinal) T-cell lymphoma (EATL; C86.2). Individuals with a recorded diagnosis of the outcome before the index event were excluded. Time-to-event was calculated from the index date to the first occurrence of the outcome, with individuals censored at their last recorded encounter or at the end of the follow-up period.

### Statistical analysis

Baseline characteristics for all groups were presented as means with standard deviations or counts with percentages. Categorical variables were compared by the χ^2^ test and continuous variables by the two-sample t-test. PSM was performed by calculating a propensity score for each patient generated by logistic regression analysis (with exposure as the dependent variable) using the Python package scikit-learn. Time-to-event analyses were performed after 1:1 propensity-score matching using Cox proportional hazards regression to estimate hazard ratios (HRs) with 95% confidence intervals (CIs). The proportional hazards assumption was assessed using Schoenfeld residuals and χ^2^ tests via the coxph function in R’s Survival package. Cases and comparators were matched on a 1:1 ratio using the greedy nearest neighbor approach with a cut-off distance of 0.1 pooled standard deviations of the logit of the propensity score. Risk ratios (RRs) were calculated using a risk comparison approach. RR estimates with 95% CIs and associated p-values were derived from z-statistics. After Bonferroni correction for multiple comparisons, p-values < 0.01 and <0.0071 were considered statistically significant for the individual CVD outcomes (n = 5) and cancer outcomes (n = 7), respectively.

### Ethics

The study was approved by the Swedish Ethical Review Authority (diary number 2025-03805-02). Individual patient informed consent was waived because the study used de-identified electronic health record data.

### Role of the funding source

Funders had no role in study design, data collection, data analysis, interpretation, writing of the report or decision to submit.

## Results

### Characteristics of the study population

Data from 129,462,076 individuals were screened. After PSM, the number of participants was 204,056 for CeD and 6896 for DH, with equal numbers of comparators for each cohort (Fig. 1, Table 1). The mean age of patients with CeD and DH was 42.7 and 53.1 years, respectively. Female patients constituted 70.5% of the CeD group and 53.2% of the DH group. No standardised differences (>0.001) were found in any of the retained matching variables after PSM. Mean follow-up time ranged 4.7–4.9 years across all primary analyses. The mortality rates were 9.3 per 1000 person-years for patients with CeD and 7.8 for comparators, with 17.0 for patients with DH and 13.5 for their comparators. Incidence rates (IRs) for all outcomes appear in Supplementary Table S1.

**Table 1:** Baseline characteristics of study participants.

Characteristic	Definition (ICD-10-CM)	Before matching			After matching		
		Exposed	Unexposed	Std. diff.	Exposed	Unexposed	Std. diff.
Coeliac disease							
n	–	208,244	16,633,405	–	204,056	204,056	–
Age at index (years, SD)^a^	–	42.7 ± 20.1	44.2 ± 20.9	0.077	42.7 ± 20.1	42.7 ± 20.1	<0.001
Black or African American (%)	–	3.1	13.0	0.369	3.1	13.1	0.376
White (%)	–	81.3	63.7	0.401	81.3	64.5	0.384
Hispanic or Latino (%)	–	4.3	8.2	0.161	4.3	8.7	0.182
Female (%)^a^	–	70.5	53.1	0.364	70.5	70.5	<0.001
Male (%)	–	29.5	46.9	0.364	29.5	29.5	<0.001
Nicotine dependence (%)^a^	F17	4.7	6.0	0.057	4.7	4.7	<0.001
Essential (primary) hypertension (%)^a^	I10	15.3	21.2	0.152	15.3	15.3	<0.001
Diabetes mellitus (%)^a^	E08–E13	8.7	8.7	0.002	8.7	8.7	<0.001
Overweight and obesity (%)	E66	9.0	9.5	0.016	9.0	9.3	0.010
Chronic kidney disease (%)	N18	2.5	3.2	0.046	2.5	2.3	0.013
Chronic obstructive pulmonary disease (%)	J44	2.5	3.2	0.030	2.3	2.0	0.019
Disorders of lipoprotein metabolism and other lipidemias (%)	E78	16.0	19.4	0.090	16.0	15.8	0.006
Alcohol related disorders (%)	F10	1.5	1.8	0.025	1.5	1.4	0.012
Persons with potential health hazards related to socioeconomic and psychosocial circumstances (%)	Z55–Z65	1.6	1.7	0.004	1.6	1.7	0.004
Immunosuppressant medication (%)	ATC: L04	3.4	1.6	0.117	3.4	1.6	0.115
Dermatitis herpetiformis							
n	–	7809	16,632,358	–	6896	6896	–
Age at index (years, SD)^a^	–	53.1 ± 18.7	44.2 ± 20.9	0.449	53.1 ± 18.7	53.1 ± 18.7	<0.001
Black or African American (%)	–	5.8	13.0	0.250	5.8	11.5	0.206
White (%)	–	75.4	63.7	0.257	75.4	66.8	0.190
Hispanic or Latino (%)	–	4.7	8.2	0.141	4.7	6.7	0.086
Female (%)^a^	–	53.2	53.1	0.003	53.2	53.2	<0.001
Male (%)	–	46.8	46.9	0.003	46.8	46.8	<0.001
Nicotine dependence (%)^a^	F17	8.4	6.0	0.093	8.4	8.4	<0.001
Essential (primary) hypertension (%)^a^	I10	28.9	21.2	0.179	28.9	28.9	<0.001
Diabetes mellitus (%)^a^	E08–E13	13.4	8.7	0.151	13.4	13.4	<0.001
Overweight and obesity (%)	E66	13.9	9.5	0.138	13.9	11.9	0.060
Chronic kidney disease (%)	N18	7.1	3.2	0.176	7.1	4.3	0.121
Chronic obstructive pulmonary disease (%)	J44	5.6	2.7	0.145	5.6	4.3	0.063
Disorders of lipoprotein metabolism and other lipidemias (%)	E78	27.0	19.4	0.180	27.0	26.9	0.002
Alcohol related disorders (%)	F10	2.5	1.8	0.044	2.5	2.4	0.007
Persons with potential health hazards related to socioeconomic and psychosocial circumstances (%)	Z55–Z65	2.5	1.7	0.056	2.5	2.1	0.027
Immunosuppressant medication (%)	ATC: L04	5.9	1.6	0.231	5.9	1.9	0.207

ICD-10-CM: International Classification of Diseases, 10th edition, clinical modification; SD: standard deviation; Std. diff.: standardised difference.

a Variable included in the propensity-score matching.

### Health outcomes in coeliac disease

**Fig. 2:**
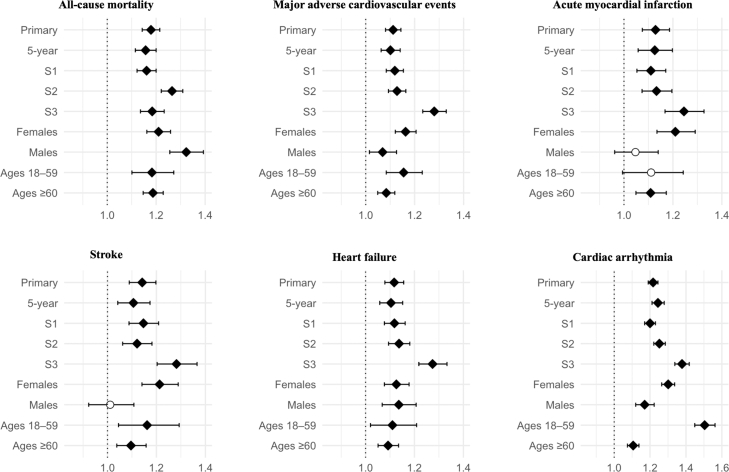
Risk of mortality and cardiovascular disease in patients with coeliac disease. Forest plots showing adjusted hazard ratios (aHRs) and 95% confidence intervals for all analyses: primary (unrestricted follow-up), 5-year follow-up (time-restricted), S1 (excluding outcomes within the first 6 months of follow-up), S2 (implementing a required healthcare encounter ≥ 6 months before index), S3 (cohort definition with ≥2 codes of coeliac disease required), and subgroups stratified by sex (female, male) and age (18–59, ≥60). The vertical dotted line indicates a null aHR of 1.0. Filled diamonds denote statistically significant associations and unfilled circles indicate non-significant results.

Patients with CeD had a modestly increased risk of all-cause mortality (aHR 1.18; 95% CI 1.14–1.22). Also, MACE (aHR 1.11; 1.08–1.14), as well as its components MI, stroke, heart failure, and cardiac arrhythmia (aHRs ranged from 1.11 to 1.22), were increased in CeD (Fig. 2). Results remained consistent across the time-restricted analysis and all sensitivity analyses. Moreover, the risk magnitudes were higher in males and individuals aged ≥ 60 (although the 95% CIs for the latter subgroup strata overlapped) (Supplementary Table S2).

**Fig. 3:**
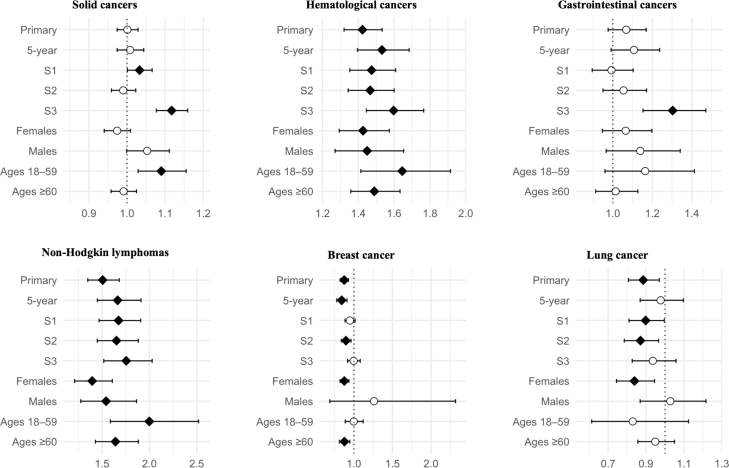
Cancer risk among individuals with coeliac disease. Forest plots showing adjusted hazard ratios (aHRs) and 95% confidence intervals for all analyses: primary (unrestricted follow-up), 5-year follow-up (time-restricted), S1 (excluding outcomes within the first 6 months of follow-up), S2 (implementing a required healthcare encounter ≥6 months before index), S3 (cohort definition with ≥2 codes of coeliac disease required), and subgroups stratified by sex (female, male) and age (18–59, ≥60). The vertical dotted line indicates a null aHR of 1.0. Filled diamonds indicate statistically significant associations, and unfilled circles indicate non-significant results.

CeD was also associated with several cancers. The risk of haematological cancer was notably elevated (aHR 1.42; 1.32–1.53), including non-Hodgkin lymphoma (aHR 1.51; 1.35–1.68). Breast (aHR 0.87) and lung cancer (0.89) risks were significantly decreased, whereas no risk difference between CeD and comparators was observed for solid cancers (aHR 1.00; 0.97–1.03) or GI cancers (aHR 1.07; 0.98–1.17) (Fig. 3). Results were largely consistent across sensitivity analyses (Supplementary Table S3). Regarding EATL, no outcomes were observed among comparators and therefore the relative risk could not be calculated. Nevertheless, by one estimate, the HR would have exceeded 21 even if assuming one case of EATL among comparators. The aHRs of the cancer types which were positively associated with CeD were even higher in males and individuals aged 18–59 years (although the 95% CIs were overlapping across the strata of both subgroups, Supplementary Table S3). The falsification endpoint (varicose veins) showed no significant association with CeD, supporting the robustness of the findings (Supplementary Table S4).

### Health outcomes in dermatitis herpetiformis

**Fig. 4:**
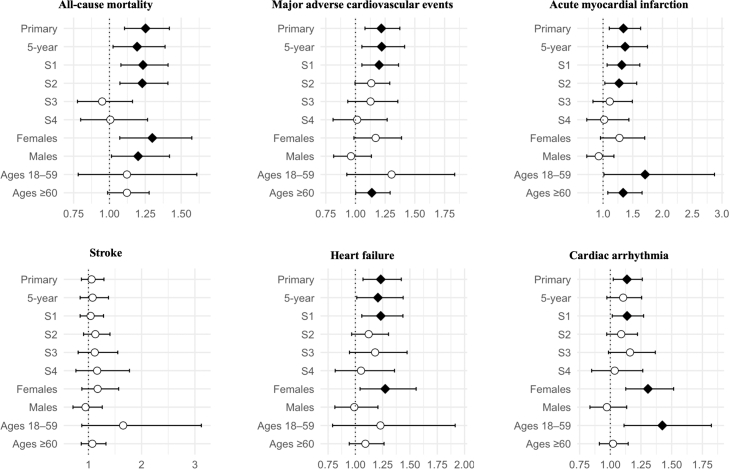
Risk of mortality and cardiovascular disease in patients with dermatitis herpetiformis. Forest plots showing adjusted hazard ratios (aHRs) and 95% confidence intervals for all analyses: primary (unrestricted follow-up), 5-year follow-up (time-restricted), S1 (excluding outcomes within the first 6 months of follow-up), S2 (implementing a required healthcare encounter ≥6 months before index), S3 (cohort definition with ≥2 codes of dermatitis herpetiformis required), S4 (cohort definition with required dapsone treatment), and subgroups stratified by sex (female, male) and age (18–59, ≥60). The vertical dotted line indicates a null aHR of 1.0. Filled diamonds denote statistically significant associations and unfilled circles denote non-significant results.

Patients with DH exhibited an increased mortality risk (aHR 1.25; 1.11–1.42, p < 0.001). However, in the two sensitivity analyses using stricter definitions for DH, the mortality risk in DH did not significantly differ from that of comparators. We found significant risk elevations for MACE and specifically for MI, heart failure, and cardiac arrhythmia, but not for stroke (Fig. 4). Overall, risk associations persisted in sensitivity analyses, except when the cohort was defined by both DH and documented dapsone treatment (S4), where no associations were observed for CVD outcomes (Supplementary Table S5). Elevated mortality risk was slightly higher in females than males. Individuals aged 18–59 exhibited higher aHRs than those aged ≥ 60 years for all CVD-related outcomes (Supplementary Table S5).

**Fig. 5:**
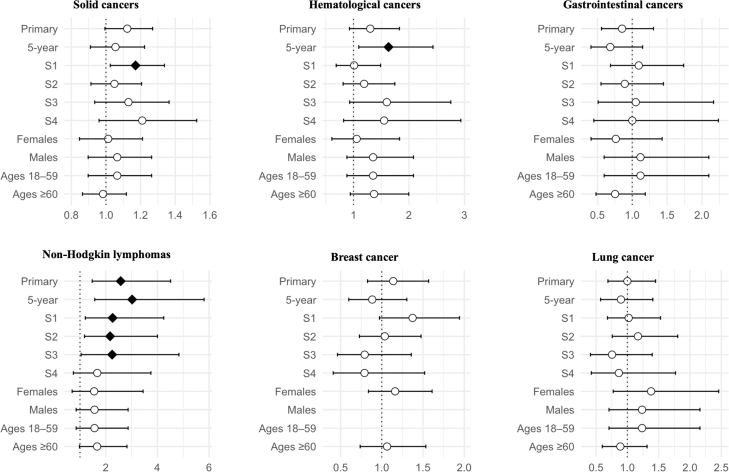
Risk of cancer in patients with dermatitis herpetiformis. Forest plots showing adjusted hazard ratios (aHRs) and 95% confidence intervals for all analyses: primary (unrestricted follow-up), 5-year follow-up (time-restricted), S1 (excluding outcomes within the first 6 months of follow-up), S2 (implementing a required healthcare encounter ≥6 months before index), S3 (cohort definition with ≥2 codes of dermatitis herpetiformis required), S4 (cohort definition with required dapsone treatment), and subgroups stratified by sex (female, male) and age (18–59, ≥60). The vertical dotted line indicates a null aHR of 1.0. Filled diamonds indicate statistically significant associations and unfilled circles indicate non-significant results. Statistics could not be calculated for breast cancer in the male and 18–59 subgroups due to insufficient number of events.

Cancer risk estimates were generally less pronounced in DH than in CeD. A significantly increased risk of non-Hodgkin lymphoma was, however, observed (aHR 2.58; 1.47–4.51), which remained consistent across all sensitivity analyses (Fig. 5, Supplementary Table S6). EATL was also analysed, but no outcomes were recorded in either group. The falsification endpoint showed no significant association with DH, suggesting the robustness of the findings.

Additionally, for both CeD and DH, additional sensitivity analyses (i) including race/ethnicity in the PSM and (ii) excluding prior IBD and GI cancers yielded comparable findings (Supplementary Table S7).

## Discussion

This large U.S. cohort study found that CeD and DH are both associated with modest but statistically significant increased risks of mortality and CVD, including MACE. Although the relative risk increases were generally small (aHRs approximately 1.1–1.2 for several outcomes in CeD), the high prevalence of CeD suggests that even modest elevations may have meaningful population-level implications. However, risks for specific cancer types differed between the two conditions: CeD was linked to haematological malignancies (including non-Hodgkin lymphoma) but showed reduced risks for breast and lung cancers, whereas DH was exclusively associated with non-Hodgkin lymphoma. This new and large-scale evidence contributes to a field that has been limited in scope, and adds non-European insights into CeD- and DH-associated mortality. In order to isolate the direct association between outcomes and DH without competing risk from CeD, we excluded individuals with prior CeD diagnoses from the DH cohort to allow independent evaluation of these phenotypes; however, this approach may limit comparability with studies that include DH within broader CeD populations. In addition, death may have acted as a competing event for non-fatal outcomes, and because competing-risk analyses were not performed, these estimates should be interpreted with some caution.

With data up to 2025, we observed an 18% increased mortality risk in CeD. This rate is similar to most, though not all,^4^ previous risk estimates (1.14, 1.29 (diagnosed CeD)).^2^^,^^5^ To our knowledge, this is the first U.S. population-based study on mortality in diagnosed CeD. Our results on MACE confirm earlier findings,^6^, ^7^, ^8^ and augment current evidence by incorporating nicotine dependence and more comorbidities in the statistical models, while also examining CeD-related mortality in a non-European setting.

Our results support previous findings of a decreased risk of breast and lung cancer in CeD.^10^ Of note, we only observed decreased risk of breast cancer in individuals ≥60 years of age. This is possibly due to a lower body mass index (protective against breast cancer) and reduced smoking prevalence in patients with CeD.^31^ Higher risks of non-Hodgkin lymphoma and haematological cancers were identified in CeD, but not of solid cancers. The absolute risk of any haematological cancer was below 1%. Marginally higher risk magnitudes for haematological malignancies were seen in females (vs. males) and individuals below the age of 60 (vs. those ≥60 years). The lack of EATL events among comparators prevented the calculation of relative risk estimates for this cancer in CeD. However, assuming that there had been one case of EATL among comparators, the minimum HR for future EATL in CeD would have exceeded 21. During follow-up, only 1 in 10,000 patients with CeD developed EATL. Considering earlier reports,^32^^,^^33^ the lack of an association between CeD and GI cancer in our study may seem surprising. Nevertheless, recent population-based studies show a limited increase of GI cancer risk beyond the first year,^10^ with surveillance/detection bias (i.e., the diagnosis of CeD during early cancer symptom assessment) potentially accounting for part of observed association.

The mortality rate among those with DH was 8.36% during a mean follow-up of 4.9 years, as opposed to 6.54% in matched comparators, yielding an aHR of 1.25 (95% CI, 1.10–1.42). The slightly older age of the DH population compared with the CeD population (mean ages 53.1 and 42.7 years, respectively) may have contributed to the higher absolute risk observed. However, the aHR for mortality was not increased in patients with ≥2 diagnostic codes for DH or in DH treated with dapsone. Therefore, caution is warranted when interpreting our mortality findings in DH. This also suggests that some degree of exposure misclassification may be present in the primary DH cohort. These findings highlight the importance of cautious interpretation when using large and heterogeneous registries.

Notably, our data also indicates a significantly increased risk of MACE in DH, with an aHR of 1.22 (1.08–1.37). This estimate exceeded that observed in the CeD analysis (aHR 1.11). This finding diverges from that of Nilsson et al. (2023), who analysed a much smaller Finnish DH sample and found no increase for CVD.^22^ Variations in adherence to a GFD and population risk profiles (e.g., comorbidities and ethnicities) may account for this discrepancy.

Concerning cancer risk, our findings confirm a significantly elevated risk of non-Hodgkin lymphoma in DH (aHR 2.58 in our study), particularly during the first five years of DH diagnosis. This increase aligns with previous research.^25^ Notably, our HR is appreciably lower than that of Collin et al. (1996).^16^ The lower HR in our study may reflect improvements in DH diagnosis and management, or inclusion of patients with DH from non-tertiary settings who were generally healthier. Of note, the Collin et al. study was also small, and the expected number of non-Hodgkin lymphoma cases was 0.4 in their study; hence, four events in that study constituted a 10-fold increase.^16^

These findings have potential implications for clinical care. Given the modest but consistent elevation in cardiovascular risk observed in CeD and possibly DH, clinicians may consider heightened attention to smoking cessation, blood pressure control, and lipid monitoring. The increased risk of non-Hodgkin lymphoma is rare in absolute risk terms, but nonetheless highlights the importance of evaluating persistent systemic symptoms such as unexplained weight loss, lymphadenopathy, or refractory gastrointestinal symptoms. Further research is needed to determine if systematic workup would be beneficial in these cohorts. From a public health perspective, the high prevalence of CeD suggests that even modest increases in cardiometabolic risk could translate into meaningful population-level disease burden.

We conducted PSM not only for age and sex but also for nicotine dependence and relevant comorbidities. However, notable crude differences were observed. First, there is a reduced incidence of chronic obstructive pulmonary disease in patients with CeD after matching, likely suggesting an inverse association with smoking (despite our efforts to match for nicotine dependence).^31^ Second, we observed a lower rate of primary hypertension, consistent with findings that CVD risk factors in patients with CeD may differ from the general population.^34^ In contrast, patients with DH exhibited higher comorbidity than those with CeD, which could suggest modest selection bias. Nevertheless, the TriNetX U.S. network has been reported to closely resemble the U.S. population based on census data,^35^ supporting the generalisability of our findings.

A major strength of this study is the large number of participants with CeD and DH. The substantial cohorts of over 200,000 individuals with CeD and nearly 7000 with DH facilitated important subgroup analyses. The large dataset further enabled the examination of specific CVDs and cancer outcomes. Further, we implemented several sensitivity analyses and a falsification endpoint to test the study’s robustness. The use of common pathologies as falsification endpoints without known epidemiological association with CeD or DH provided reassurance that major residual confounding or systematic information bias was unlikely to explain the observed associations.

This study has some limitations. Misclassification and data entry errors cannot be excluded, and we lacked access to serologic or histologic data to confirm the diagnosis of CeD or DH. To our knowledge, there has been no validation of CeD or DH within TriNetX. TriNetX does undergo data quality checks, however.^27^ While we adjusted for numerous confounders, we cannot rule out some residual confounding. Although sensitivity analyses incorporating race/ethnicity into PSM yielded comparable findings, we did not perform outcome analyses stratified by race or ethnicity. We lacked information on physical activity and healthcare-seeking patterns, which may differ between patients with CeD or DH and the general population. Still, most of our outcomes (death, CVD, cancer) require hospital contact, suggesting a low risk of differential misclassification for the outcomes. We cannot rule out that surveillance bias may have impacted our CVD and cancer findings. However, excluding individuals with a positive outcome within 6 months of the index date limits most early detection bias. Due to computational constraints from exceedingly large cohort sizes, comparators were not sourced from the general population but rather from a cohort of individuals with general healthcare contact. This could potentially introduce “healthy user” bias if these individuals are more health-conscious and have a lower baseline risk than the general population, whereas other individuals with frequent healthcare encounters may have greater underlying comorbidity than the general population. However, the opposite is also possible; individuals in excellent health who rarely require medical attention would not appear in the dataset. Lastly, the lack of dietary data restricts our ability to assess the role of GFD adherence, particularly concerning the observed differences between CeD and DH. This is particularly relevant when interpreting differences between CeD and DH, as adherence to a strict GFD is often higher among patients with DH due to the rapid cutaneous response to gluten exposure. Differences in dietary adherence may therefore partly explain discrepancies between our findings and those reported in European DH cohorts.

### Conclusion

Our study adds contemporary large-scale evidence from a federated electronic health record network across multiple U.S. healthcare systems. By adjusting for key confounders such as age, sex, nicotine dependence, and comorbidities, using 1:1 PSM, implementing several sensitivity analyses and a falsification endpoint, our findings provide more precise and generalisable estimates compared to preceding research. Nonetheless, the elevated risk of mortality and CVD in CeD and DH warrants further inquiry, particularly into the role of dietary adherence and risk factor modification in routine clinical care. While CeD and DH share overlapping risks, especially increased mortality, their differing comorbidity patterns underscore the need for disease-specific clinical management strategies.

In conclusion, this U.S.-based retrospective cohort study of more than 200,000 patients with CeD and nearly 7000 patients with DH revealed an elevated mortality risk. CVD was associated with both CeD and DH; conversely, the elevated cancer risk was restricted to haematological malignancies for CeD and more specifically, to non-Hodgkin lymphoma for DH.

## Contributors

Conception and design: PC and JFL. Acquisition of data: PC. Statistical analysis: PC. Interpretation of data: All authors. Drafting the manuscript: PC, JFL, and JD. Critical revision for intellectual content: All authors. All authors have read and approved the manuscript. PC and RJL accessed and verified the data.

## Data sharing statement

The data used in this study were obtained from TriNetX under licence and are not publicly available. Access to the underlying data may be available to qualified researchers through TriNetX, subject to relevant permissions, licensing agreements, and platform access requirements.

## Use of artificial intelligence

No generative artificial intelligence was used to generate the data, perform the analyses, or draw the conclusions of this study. Generative artificial intelligence was used only for limited language editing and manuscript preparation, with full human review and responsibility retained by the authors.

## Declaration of interests

JFL has coordinated an unrelated study on behalf of the Swedish IBD quality register (SWIBREG). That study received funding from the Janssen Corporation. JFL has received financial support from Merck/MSD for an unrelated study on IBD and for developing a paper reviewing national healthcare registers in China. JFL also has an ongoing research collaboration on celiac disease with Takeda. RJL has received travel grants from TriNetX and research support from Novartis and Sanofi. BL has consulted for Teva Pharmaceuticals and Dualitas Therapeutics. PHRG has consulted for J&J, Takeda, and DBV technologies. All other authors have no relevant conflicts of interest to declare.
