# Supplementary material for: Mortality, cardiovascular disease, and cancer in coeliac disease and dermatitis herpetiformis: a matched cohort study
Source: Lancet Reg Health Am. 2026 May 28;60:101512. doi: 10.1016/j.lana.2026.101512 (PMC13235495; doi:10.1016/j.lana.2026.101512)
Supplement: Supplementary Tables S1–S7 [file mmc1.pdf]

SUPPLEMENTARY MATERIAL

Supplemental methods

Data sources: TriNetX data include aggregated information on demographics, diagnoses (coded using the *International Classification of Diseases, Tenth Revision, Clinical Modification* [ICD-10-CM]), medical procedures (categorized under the *International Classification of Diseases, Tenth Revision Procedure Coding System*, or *Current Procedural Terminology*), prescribed medications (identified through the *Veterans Affairs Drug Classification System* and *RxNorm* codes), laboratory tests (classified using the *Logical Observation Identifiers Names and Codes*), and records of healthcare utilization.

Supplementary table 1. **Incidence rates for all outcomes in celiac disease (CeD) and dermatitis herpetiformis (DH), primary analysis.**
The mean follow-up lengths for CeD patients were 4.7 years, CeD comparators 5.0 years, DH patients 4.9 years, and DH comparators 4.8 years.

Outcome	Exposed cohort (cases)			Unexposed cohort (comparators)		
	N total	N outcomes	Incidence rate (per 1,000 person-years)	N total	N outcomes	Incidence rate (per 1,000 person-years)
**Celiac disease (primary analysis)**						
All-cause mortality	200551	8791	9.3	201285	7841	7.8
Major adverse cardiovascular events	195428	9795	10.6	196920	9360	9.5
Acute myocardial infarction	201602	3280	3.4	202182	3079	3.1
Stroke	201359	3517	3.7	201677	3247	3.2
Heart failure	198983	6690	7.1	199793	6334	6.4
Cardiac arrhythmia	187322	16289	18.4	192953	14592	15.2
Solid cancers	191608	9621	10.6	194063	10191	10.5
Hematological cancers	202008	1603	1.7	202722	1188	1.2
Gastrointestinal cancers	202945	955	1.0	203244	934	0.9
Non-Hodgkin lymphomas	203109	769	0.8	203537	537	0.5
Breast cancer	201257	1872	2.0	201405	2251	2.2
Lung cancer	203498	838	0.9	203513	992	1.0
EATL	204037	20	<0.1	204056	0	0
**Dermatitis herpetiformis (primary analysis)**						
All-cause mortality	6768	566	17.0	6770	443	13.5
Major adverse cardiovascular events	6328	587	18.8	6405	488	15.7
Acute myocardial infarction	6717	238	7.2	6752	176	5.4
Stroke	6748	202	6.1	6751	188	5.7
Heart failure	6499	426	13.3	6585	348	10.9
Cardiac arrhythmia	6068	742	24.8	6299	678	22.2
Solid cancers	6188	536	17.6	6310	485	15.9
Hematological cancers	6764	77	2.3	6831	59	1.8

Gastrointestinal cancers	6840	40	1.2	6839	46	1.4
Non-Hodgkin lymphomas	6822	44	1.3	6871	17	0.5
Breast cancer	6774	81	2.4	6763	70	2.1
Lung cancer	6848	56	1.7	6850	55	1.7

Supplementary table 2. **Detailed results of all-cause mortality and cardiovascular disease outcomes in celiac disease.**
Risk of all-cause mortality, major adverse cardiovascular events, acute myocardial infarction, stroke, heart failure, and cardiac arrhythmia in individuals with celiac disease. P-values <0.01 were considered statistically significant after Bonferroni correction for the five cardiovascular outcomes.
CI: confidence interval.

Outcome	Exposed cohort (celiac disease)			Unexposed cohort (comparators)			Hazard ratio (95% CI)	P-value	Risk ratio (95% CI)	P-value
	N total	N outcomes	Risk (%)	N total	N outcomes	Risk (%)				
**Unrestricted follow-up (primary analysis)**										
All-cause mortality	200551	8791	4.38	201285	7841	3.90	1.179 (1.143, 1.215)	<0.001	1.125 (1.092, 1.159)	<0.001
Major adverse cardiovascular events	195428	9795	5.01	196920	9360	4.75	1.112 (1.081, 1.144)	<0.001	1.054 (1.026, 1.084)	<0.001
Acute myocardial infarction	201602	3280	1.63	202182	3079	1.52	1.129 (1.075, 1.186)	<0.001	1.068 (1.017, 1.122)	0.008
Stroke	201359	3517	1.75	201677	3247	1.61	1.142 (1.089, 1.198)	<0.001	1.085 (1.035, 1.137)	0.001
Heart failure	198983	6690	3.36	199793	6334	3.17	1.117 (1.079, 1.156)	<0.001	1.061 (1.025, 1.097)	0.001
Cardiac arrhythmia	187322	16289	8.70	192953	14592	7.56	1.217 (1.190, 1.244)	<0.001	1.150 (1.125, 1.175)	<0.001
**5-year follow-up (time-restricted analysis)**										
All-cause mortality	200551	5973	2.98	201285	5281	2.62	1.157 (1.115, 1.200)	<0.001	1.135 (1.094, 1.177)	<0.001
Major adverse cardiovascular events	195428	6470	3.31	196920	6031	3.06	1.101 (1.063, 1.141)	<0.001	1.081 (1.044, 1.119)	<0.001
Acute myocardial infarction	201602	2102	1.04	202182	1909	0.94	1.126 (1.058, 1.198)	<0.001	1.104 (1.038, 1.174)	0.002
Stroke	201359	2277	1.13	201677	2101	1.04	1.106 (1.042, 1.174)	0.001	1.085 (1.023, 1.151)	0.006
Heart failure	198983	4395	2.21	199793	4072	2.04	1.104 (1.058, 1.152)	<0.001	1.084 (1.039, 1.130)	<0.001
Cardiac arrhythmia	187322	11347	6.06	192953	9624	4.99	1.244 (1.211, 1.279)	<0.001	1.214 (1.183, 1.247)	<0.001
**Outcomes during the first six months excluded (sensitivity analysis S1)**										
All-cause mortality	188525	7185	3.81	189902	6554	3.45	1.161 (1.122, 1.200)	<0.001	1.104 (1.069, 1.141)	<0.001
Major adverse cardiovascular events	183862	8067	4.39	185363	7704	4.16	1.119 (1.084, 1.154)	<0.001	1.056 (1.024, 1.088)	0.001
Acute myocardial infarction	190701	2777	1.46	191271	2657	1.39	1.110 (1.053, 1.171)	<0.001	1.048 (0.994, 1.105)	0.080
Stroke	190482	2898	1.52	190905	2679	1.40	1.147 (1.088, 1.209)	<0.001	1.084 (1.029, 1.142)	0.002
Heart failure	187635	5534	2.95	188417	5263	2.79	1.118 (1.077, 1.162)	<0.001	1.056 (1.017, 1.096)	0.004
Cardiac arrhythmia	174815	13110	7.50	180587	12015	6.65	1.200 (1.170, 1.230)	<0.001	1.127 (1.101, 1.154)	<0.001

**Implementing a required healthcare encounter ≥6 months before index (sensitivity analysis S2)**										
All-cause mortality	135906	6866	5.05	136756	5891	4.31	1.265 (1.221, 1.309)	<0.001	1.173 (1.134, 1.213)	<0.001
Major adverse cardiovascular events	132086	7727	5.85	133163	7508	5.64	1.128 (1.093, 1.164)	<0.001	1.038 (1.006, 1.070)	0.019
Acute myocardial infarction	137223	2695	1.96	137626	2597	1.89	1.133 (1.074, 1.196)	<0.001	1.041 (0.987, 1.098)	0.142
Stroke	137069	2757	2.01	137370	2674	1.95	1.121 (1.062, 1.182)	<0.001	1.033 (0.980, 1.089)	0.223
Heart failure	134962	5380	3.99	135547	5158	3.81	1.137 (1.094, 1.181)	<0.001	1.048 (1.009, 1.088)	0.015
Cardiac arrhythmia	125029	12485	9.99	129490	11300	8.73	1.252 (1.221, 1.285)	<0.001	1.144 (1.117, 1.172)	<0.001
**Cohort definition requiring ≥2 codes of celiac disease (sensitivity analysis S3)**										
All-cause mortality	105298	5243	4.98	105916	4006	3.78	1.184 (1.136, 1.233)	<0.001	1.316 (1.265, 1.371)	<0.001
Major adverse cardiovascular events	103224	6627	6.42	103636	4709	4.54	1.280 (1.233, 1.329)	<0.001	1.413 (1.362, 1.465)	<0.001
Acute myocardial infarction	106193	2241	2.11	106414	1624	1.53	1.245 (1.168, 1.327)	<0.001	1.383 (1.298, 1.473)	<0.001
Stroke	106112	2310	2.18	106150	1621	1.53	1.282 (1.203, 1.366)	<0.001	1.426 (1.339, 1.518)	<0.001
Heart failure	104978	4580	4.36	105090	3249	3.09	1.274 (1.218, 1.333)	<0.001	1.411 (1.350, 1.475)	<0.001
Cardiac arrhythmia	98824	11044	11.18	101631	7551	7.43	1.378 (1.338, 1.418)	<0.001	1.504 (1.463, 1.547)	<0.001
**Females**										
All-cause mortality	133208	5037	3.78	133944	4428	3.31	1.210 (1.162, 1.259)	<0.001	1.144 (1.099, 1.190)	<0.001
Major adverse cardiovascular events	131070	6034	4.60	131912	5572	4.22	1.163 (1.121, 1.206)	<0.001	1.090 (1.052, 1.129)	<0.001
Acute myocardial infarction	134656	1982	1.47	134938	1753	1.30	1.210 (1.135, 1.291)	<0.001	1.133 (1.063, 1.208)	<0.001
Stroke	134258	2226	1.66	134547	1961	1.46	1.213 (1.141, 1.289)	<0.001	1.138 (1.071, 1.208)	<0.001
Heart failure	133078	3980	2.99	133428	3770	2.83	1.126 (1.077, 1.178)	<0.001	1.058 (1.013, 1.106)	0.011
Cardiac arrhythmia	125434	10778	8.59	129108	9154	7.09	1.301 (1.265, 1.337)	<0.001	1.212 (1.180, 1.245)	<0.001
**Males**										
All-cause mortality	48499	3260	6.72	48776	2544	5.22	1.323 (1.256, 1.393)	<0.001	1.289 (1.226, 1.355)	<0.001
Major adverse cardiovascular events	46337	2912	6.28	46777	2826	6.04	1.069 (1.015, 1.126)	0.011	1.040 (0.989, 1.094)	0.123
Acute myocardial infarction	48297	1081	2.24	48502	1068	2.20	1.047 (0.962, 1.140)	0.285	1.016 (0.935, 1.105)	0.702
Stroke	48542	917	1.89	48584	933	1.92	1.011 (0.923, 1.108)	0.808	0.984 (0.899, 1.077)	0.721
Heart failure	47496	2154	4.54	47798	1964	4.11	1.136 (1.068, 1.207)	<0.001	1.104 (1.040, 1.172)	0.001
Cardiac arrhythmia	44382	4175	9.41	45797	3807	8.31	1.170 (1.120, 1.223)	<0.001	1.132 (1.085, 1.180)	<0.001

**Ages 18-59**										
All-cause mortality	125097	1559	1.25	125814	1382	1.10	1.183 (1.101, 1.272)	<0.001	1.135 (1.056, 1.219)	0.001
Major adverse cardiovascular events	125387	1988	1.59	125679	1812	1.44	1.155 (1.084, 1.231)	<0.001	1.100 (1.032, 1.171)	0.003
Acute myocardial infarction	126729	634	0.50	126766	601	0.47	1.111 (0.994, 1.242)	0.064	1.055 (0.944, 1.179)	0.344
Stroke	126513	712	0.56	126636	644	0.51	1.162 (1.045, 1.293)	0.006	1.107 (0.995, 1.231)	0.062
Heart failure	126240	1094	0.87	126347	1034	0.82	1.110 (1.020, 1.209)	0.016	1.059 (0.973, 1.152)	0.185
Cardiac arrhythmia	121259	6494	5.36	123615	4656	3.77	1.504 (1.449, 1.562)	<0.001	1.422 (1.370, 1.475)	<0.001
**Ages ≥60**										
All-cause mortality	64865	7062	10.89	64982	6079	9.35	1.187 (1.147, 1.229)	<0.001	1.164 (1.127, 1.202)	<0.001
Major adverse cardiovascular events	59836	7439	12.43	60896	7164	11.76	1.084 (1.049, 1.119)	<0.001	1.057 (1.025, 1.089)	<0.001
Acute myocardial infarction	64390	2560	3.98	64870	2386	3.68	1.109 (1.049, 1.173)	<0.001	1.081 (1.023, 1.142)	0.005
Stroke	64422	2638	4.09	64646	2477	3.83	1.096 (1.038, 1.158)	0.001	1.069 (1.013, 1.128)	0.015
Heart failure	62322	5366	8.61	62934	5081	8.07	1.092 (1.051, 1.135)	<0.001	1.066 (1.028, 1.106)	0.001
Cardiac arrhythmia	56112	9171	16.34	58938	8935	15.16	1.106 (1.074, 1.138)	<0.001	1.078 (1.050, 1.107)	<0.001

Supplementary table 3. **Detailed results of all cancer outcomes in celiac disease.**
Risk of solid cancers, hematological cancers, gastrointestinal cancers, non-Hodgkin lymphomas, breast cancer, lung cancer, and enteropathy-associated T-cell lymphoma in individuals with celiac disease. P-values <0.0071 were considered statistically significant after Bonferroni correction for seven outcomes.
CI: confidence interval; EATL: Enteropathy-associated T-cell lymphoma.

Outcome	Exposed cohort (celiac disease)			Unexposed cohort (comparators)			Hazard ratio (95% CI)	P-value	Risk ratio (95% CI)	P-value
	N total	N outcomes	Risk (%)	N total	N outcomes	Risk (%)				
**Unrestricted follow-up (primary analysis)**										
Solid cancers	191608	9621	5.02	194063	10191	5.25	1.001 (0.974, 1.029)	0.941	0.956 (0.931, 0.982)	0.001
Hematological cancers	202008	1603	0.79	202722	1188	0.59	1.423 (1.320, 1.533)	<0.001	1.354 (1.257, 1.459)	<0.001
Gastrointestinal cancers	202945	955	0.47	203244	934	0.46	1.068 (0.976, 1.169)	0.154	1.024 (0.936, 1.120)	0.606
Non-Hodgkin lymphomas	203109	769	0.38	203537	537	0.26	1.506 (1.349, 1.682)	<0.001	1.435 (1.285, 1.602)	<0.001
Breast cancer	201257	1872	0.93	201405	2251	1.12	0.874 (0.822, 0.929)	<0.001	0.832 (0.783, 0.885)	<0.001
Lung cancer	203498	838	0.41	203513	992	0.49	0.885 (0.807, 0.970)	0.009	0.845 (0.771, 0.926)	<0.001
EATL	204037	20	0.01	204056	0	0.00	-	-	-	-
**5-year follow-up (time-restricted analysis)**										
Solid cancers	181879	6186	3.40	184030	6349	3.45	1.008 (0.974, 1.044)	0.644	0.986 (0.953, 1.020)	0.417
Hematological cancers	191568	1079	0.56	192162	723	0.38	1.532 (1.395, 1.684)	<0.001	1.497 (1.363, 1.645)	<0.001
Gastrointestinal cancers	192412	652	0.34	192692	602	0.31	1.107 (0.991, 1.237)	0.072	1.085 (0.971, 1.211)	0.15
Non-Hodgkin lymphomas	192592	530	0.28	192918	326	0.17	1.664 (1.450, 1.911)	<0.001	1.629 (1.419, 1.869)	<0.001
Breast cancer	190862	1117	0.59	190881	1359	0.71	0.841 (0.777, 0.910)	<0.001	0.822 (0.760, 0.889)	<0.001
Lung cancer	192936	554	0.29	192932	581	0.30	0.977 (0.869, 1.097)	0.693	0.954 (0.849, 1.071)	0.422
EATL	193449	17	0.01	193465	0	0.00	-	-	-	-
**Outcomes during the first six months excluded (sensitivity analysis S1)**										
Solid cancers	180469	7683	4.26	182572	7943	4.35	1.033 (1.001, 1.066)	0.041	0.979 (0.949, 1.009)	0.166
Hematological cancers	191258	1227	0.64	191994	884	0.46	1.474 (1.352, 1.608)	<0.001	1.393 (1.278, 1.519)	<0.001
Gastrointestinal cancers	192187	680	0.35	192544	723	0.38	0.993 (0.895, 1.103)	0.901	0.942 (0.849, 1.046)	0.265
Non-Hodgkin lymphomas	192432	578	0.30	192832	366	0.19	1.674 (1.468, 1.908)	<0.001	1.583 (1.389, 1.804)	<0.001
Breast cancer	190632	1529	0.80	190560	1705	0.89	0.947 (0.884, 1.015)	0.121	0.896 (0.837, 0.960)	0.002

Lung cancer	192814	668	0.35	192813	786	0.41	0.898 (0.810, 0.996)	0.041	0.850 (0.767, 0.942)	0.002
EATL	193438	10*	0.01	193465	0	0.00	-	-	-	-
**Implementing a required healthcare encounter ≥6 months before index (sensitivity analysis S2)**										
Solid cancers	129131	7059	5.47	131021	7804	5.96	0.990 (0.959, 1.023)	0.559	0.918 (0.890, 0.947)	<0.001
Hematological cancers	137693	1179	0.86	138238	873	0.63	1.466 (1.343, 1.600)	<0.001	1.356 (1.243, 1.479)	<0.001
Gastrointestinal cancers	138417	697	0.50	138626	711	0.51	1.054 (0.950, 1.171)	0.320	0.982 (0.885, 1.090)	0.730
Non-Hodgkin lymphomas	138559	567	0.41	138854	372	0.27	1.652 (1.449, 1.883)	<0.001	1.527 (1.340, 1.740)	<0.001
Breast cancer	136934	1392	1.02	137087	1681	1.23	0.896 (0.835, 0.962)	0.003	0.829 (0.772, 0.890)	<0.001
Lung cancer	138835	645	0.46	138871	799	0.58	0.871 (0.785, 0.966)	0.009	0.807 (0.728, 0.896)	<0.001
EATL	139280	11	0.01	139291	0	0.00	-	-	-	-
**Cohort definition requiring ≥2 codes of celiac disease (sensitivity analysis S3)**										
Solid cancers	101080	6333	6.27	102263	5172	5.06	1.117 (1.077, 1.159)	<0.001	1.239 (1.195, 1.284)	<0.001
Hematological cancers	106317	1061	1.00	106685	601	0.56	1.597 (1.445, 1.765)	<0.001	1.772 (1.603, 1.957)	<0.001
Gastrointestinal cancers	106819	635	0.59	106951	440	0.41	1.303 (1.153, 1.471)	<0.001	1.445 (1.280, 1.631)	<0.001
Non-Hodgkin lymphomas	106924	536	0.50	107112	276	0.26	1.755 (1.518, 2.029)	<0.001	1.945 (1.683, 2.249)	<0.001
Breast cancer	105932	1192	1.13	106030	1074	1.01	0.996 (0.917, 1.081)	0.921	1.111 (1.023, 1.206)	0.012
Lung cancer	107121	516	0.48	107140	495	0.46	0.936 (0.827, 1.058)	0.29	1.043 (0.922, 1.179)	0.506
EATL	107370	19	0.02	107380	0	0.00	-	-	-	-
**Females**										
Solid cancers	128156	5920	4.62	129617	6494	5.01	0.974 (0.940, 1.009)	0.142	0.922 (0.891, 0.954)	<0.001
Hematological cancers	134723	938	0.70	135092	699	0.52	1.426 (1.293, 1.573)	<0.001	1.346 (1.220, 1.484)	<0.001
Gastrointestinal cancers	135248	559	0.41	135352	554	0.41	1.065 (0.947, 1.198)	0.292	1.010 (0.898, 1.135)	0.870
Non-Hodgkin lymphomas	135347	442	0.33	135543	336	0.25	1.395 (1.210, 1.608)	<0.001	1.317 (1.143, 1.518)	<0.001
Breast cancer	133353	1644	1.23	133458	1989	1.49	0.875 (0.820, 0.935)	<0.001	0.827 (0.775, 0.883)	<0.001
Lung cancer	135501	482	0.36	135519	610	0.45	0.839 (0.744, 0.945)	0.004	0.790 (0.701, 0.890)	<0.001
EATL	135842	10*	0.01	135847	0	0.00	-	-	-	-
**Males**										

Solid cancers	45911	2703	5.89	46636	2677	5.74	1.053 (0.999, 1.111)	0.056	1.026 (0.974, 1.080)	0.338
Hematological cancers	48649	531	1.09	48866	378	0.77	1.449 (1.270, 1.653)	<0.001	1.411 (1.237, 1.609)	<0.001
Gastrointestinal cancers	48915	302	0.62	49059	272	0.55	1.139 (0.966, 1.341)	0.120	1.114 (0.946, 1.311)	0.197
Non-Hodgkin lymphomas	48994	267	0.54	49143	178	0.36	1.542 (1.275, 1.864)	<0.001	1.505 (1.245, 1.818)	<0.001
Breast cancer	49315	23	0.05	49316	19	0.04	1.261 (0.687, 2.317)	0.453	1.211 (0.659, 2.222)	0.537
Lung cancer	49170	273	0.56	49175	273	0.56	1.028 (0.869, 1.216)	0.747	1.000 (0.846, 1.182)	0.999
EATL	49319	10*	0.02	49330	0	0.00	-	-	-	-
**Ages 18-59**										
Solid cancers	124305	2363	1.9	125181	2287	1.83	1.090 (1.029, 1.155)	0.003	1.041 (0.983, 1.101)	0.172
Hematological cancers	126545	431	0.34	126687	275	0.22	1.645 (1.414, 1.914)	<0.001	1.569 (1.349, 1.825)	<0.001
Gastrointestinal cancers	126920	218	0.17	127035	195	0.15	1.164 (0.960, 1.413)	0.122	1.119 (0.923, 1.357)	0.254
Non-Hodgkin lymphomas	126886	210	0.17	126987	110	0.09	1.999 (1.587, 2.518)	<0.001	1.911 (1.517, 2.406)	<0.001
Breast cancer	126564	552	0.44	126632	584	0.46	0.997 (0.887, 1.120)	0.956	0.946 (0.842, 1.062)	0.346
Lung cancer	127072	75	0.06	127099	95	0.07	0.830 (0.613, 1.124)	0.229	0.790 (0.583, 1.069)	0.125
EATL	127139	10*	0.01	127143	0	0.00	-	-	-	-
**Ages ≥60**										
Solid cancers	57575	6730	11.69	58928	7073	12.00	0.991 (0.958, 1.025)	0.596	0.974 (0.944, 1.005)	0.098
Hematological cancers	65024	1106	1.70	65487	768	1.17	1.489 (1.358, 1.633)	<0.001	1.450 (1.324, 1.589)	<0.001
Gastrointestinal cancers	65493	687	1.05	65724	694	1.06	1.014 (0.913, 1.127)	0.790	0.993 (0.894, 1.103)	0.902
Non-Hodgkin lymphomas	65707	528	0.80	65933	331	0.50	1.641 (1.430, 1.883)	<0.001	1.601 (1.396, 1.836)	<0.001
Breast cancer	64299	1207	1.88	64344	1407	2.19	0.876 (0.811, 0.946)	0.001	0.858 (0.796, 0.926)	<0.001
Lung cancer	65865	715	1.09	65855	771	1.17	0.949 (0.857, 1.050)	0.309	0.927 (0.838, 1.026)	0.143
EATL	66311	13	0.02	66323	0	0.00	-	-	-	-

Supplementary table 4. **Falsification endpoint.**
Risk of varicose veins in patients with celiac disease or dermatitis herpetiformis.

Outcome	ICD-10-CM code	Exposed cohort			Unexposed cohort (comparators)			Hazard ratio (95% CI)	P-value
		N total	N outcomes	Risk (%)	N total	N outcomes	Risk (%)		
**Celiac disease**									
Varicose veins	I83	190741	3199	1.68	191364	3288	1.72	1.021 (0.972, 1.072)	0.406
**Dermatitis herpetiformis**									
Varicose veins	I83	6011	142	2.36	6103	139	2.28	1.026 (0.812, 1.296)	0.831

Supplementary table 5. **Detailed results of all-cause mortality and cardiovascular disease outcomes in dermatitis herpetiformis.**
Risk of all-cause mortality, major adverse cardiovascular events, acute myocardial infarction, stroke, heart failure, and cardiac arrhythmia in individuals with dermatitis herpetiformis. P-values <0.01 were considered statistically significant after Bonferroni correction for the five cardiovascular outcomes.
CI: confidence interval; DH: dermatitis herpetiformis

Outcome	Exposed cohort (DH)			Unexposed cohort (comparators)			Hazard ratio (95% CI)	P-value	Risk ratio (95% CI)	P-value
	N total	N outcomes	Risk (%)	N total	N outcomes	Risk (%)				
**Unrestricted follow-up (primary analysis)**										
All-cause mortality	6768	566	8.36	6770	443	6.54	1.252 (1.105, 1.418)	<0.001	1.278 (1.134, 1.441)	<0.001
Major adverse cardiovascular events	6328	587	9.28	6405	488	7.62	1.218 (1.080, 1.374)	0.001	1.218 (1.085, 1.366)	0.001
Acute myocardial infarction	6717	238	3.54	6752	176	2.61	1.343 (1.105, 1.633)	0.003	1.359 (1.122, 1.647)	0.002
Stroke	6748	202	2.99	6751	188	2.78	1.059 (0.868, 1.292)	0.573	1.075 (0.884, 1.307)	0.469
Heart failure	6499	426	6.55	6585	348	5.28	1.233 (1.070, 1.421)	0.004	1.240 (1.081, 1.423)	0.002
Cardiac arrhythmia	6068	742	12.23	6299	678	10.76	1.136 (1.024, 1.261)	0.016	1.136 (1.030, 1.253)	0.011
**5-year follow-up (time-restricted analysis)**										
All-cause mortality	6768	366	5.41	6770	307	4.53	1.193 (1.025, 1.388)	0.022	1.193 (1.029, 1.383)	0.019
Major adverse cardiovascular events	6328	392	6.19	6405	326	5.09	1.221 (1.054, 1.414)	0.008	1.217 (1.055, 1.404)	0.007
Acute myocardial infarction	6717	154	2.29	6752	113	1.67	1.371 (1.075, 1.748)	0.011	1.370 (1.077, 1.742)	0.010
Stroke	6748	133	1.97	6751	123	1.82	1.078 (0.844, 1.377)	0.548	1.082 (0.849, 1.379)	0.526
Heart failure	6499	274	4.22	6585	230	3.49	1.207 (1.013, 1.438)	0.035	1.207 (1.016, 1.433)	0.032
Cardiac arrhythmia	6068	482	7.94	6299	453	7.19	1.105 (0.972, 1.256)	0.127	1.105 (0.976, 1.249)	0.114
**Outcomes during the first six months excluded (sensitivity analysis S1)**										
All-cause mortality	6694	492	7.35	6716	389	5.79	1.234 (1.080, 1.409)	0.002	1.269 (1.116, 1.443)	<0.001
Major adverse cardiovascular events	6248	507	8.11	6345	428	6.75	1.199 (1.054, 1.363)	0.006	1.203 (1.063, 1.362)	0.003
Acute myocardial infarction	6686	207	3.10	6732	156	2.32	1.315 (1.068, 1.619)	0.010	1.336 (1.088, 1.640)	0.005
Stroke	6726	180	2.68	6733	170	2.52	1.041 (0.844, 1.284)	0.707	1.060 (0.862, 1.304)	0.581
Heart failure	6442	369	5.73	6538	301	4.60	1.233 (1.059, 1.436)	0.007	1.244 (1.073, 1.443)	0.004
Cardiac arrhythmia	5965	639	10.71	6204	583	9.40	1.137 (1.016, 1.272)	0.025	1.140 (1.025, 1.268)	0.016

**Implementing a required healthcare encounter ≥6 months before index (sensitivity analysis S2)**										
All-cause mortality	5003	464	9.27	5008	376	7.51	1.229 (1.073, 1.408)	0.003	1.235 (1.084, 1.407)	0.001
Major adverse cardiovascular events	4596	488	10.62	4687	443	9.45	1.133 (0.996, 1.289)	0.057	1.123 (0.994, 1.269)	0.061
Acute myocardial infarction	4956	197	3.97	4988	157	3.15	1.270 (1.030, 1.567)	0.025	1.263 (1.028, 1.552)	0.026
Stroke	4985	172	3.45	5005	153	3.06	1.130 (0.909, 1.405)	0.272	1.129 (0.911, 1.398)	0.268
Heart failure	4754	360	7.57	4841	328	6.78	1.122 (0.966, 1.304)	0.130	1.118 (0.968, 1.291)	0.130
Cardiac arrhythmia	4347	589	13.55	4545	578	12.72	1.089 (0.971, 1.222)	0.143	1.065 (0.957, 1.186)	0.245
**Cohort definition requiring ≥2 codes of dermatitis herpetiformis (sensitivity analysis S3)**										
All-cause mortality	2515	209	8.31	2506	176	7.02	0.949 (0.777, 1.161)	0.613	1.183 (0.976, 1.435)	0.087
Major adverse cardiovascular events	2403	264	10.99	2387	193	8.09	1.127 (0.935, 1.357)	0.209	1.359 (1.139, 1.621)	0.001
Acute myocardial infarction	2506	105	4.19	2503	78	3.12	1.113 (0.830, 1.493)	0.474	1.345 (1.008, 1.793)	0.043
Stroke	2515	86	3.42	2506	62	2.47	1.119 (0.807, 1.552)	0.499	1.382 (1.002, 1.907)	0.048
Heart failure	2468	191	7.74	2457	133	5.41	1.181 (0.946, 1.474)	0.141	1.430 (1.154, 1.771)	0.001
Cardiac arrhythmia	2297	342	14.89	2316	248	10.71	1.161 (0.985, 1.367)	0.074	1.390 (1.193, 1.620)	<0.001
**Dermatitis herpetiformis with dapsone treatment (sensitivity analysis S4)**										
All-cause mortality	1721	159	9.24	1718	135	7.86	1.006 (0.799, 1.266)	0.960	1.176 (0.944, 1.464)	0.148
Major adverse cardiovascular events	1603	165	10.29	1613	145	8.99	1.014 (0.811, 1.267)	0.905	1.145 (0.926, 1.416)	0.210
Acute myocardial infarction	1700	71	4.18	1716	62	3.61	1.020 (0.726, 1.435)	0.908	1.156 (0.828, 1.614)	0.395
Stroke	1723	51	2.96	1710	38	2.22	1.164 (0.765, 1.772)	0.478	1.332 (0.880, 2.016)	0.174
Heart failure	1659	129	7.78	1667	108	6.48	1.052 (0.815, 1.359)	0.696	1.200 (0.938, 1.536)	0.146
Cardiac arrhythmia	1542	206	13.36	1566	180	11.49	1.036 (0.848, 1.265)	0.732	1.162 (0.964, 1.402)	0.115
**Females**										
All-cause mortality	3604	237	6.58	3629	187	5.15	1.299 (1.072, 1.574)	0.007	1.276 (1.059, 1.537)	0.010
Major adverse cardiovascular events	3421	280	8.18	3462	251	7.25	1.170 (0.987, 1.388)	0.070	1.129 (0.958, 1.330)	0.146
Acute myocardial infarction	3602	104	2.89	3614	84	2.32	1.276 (0.957, 1.702)	0.096	1.242 (0.935, 1.650)	0.133
Stroke	3595	97	2.70	3608	85	2.36	1.173 (0.876, 1.569)	0.284	1.145 (0.859, 1.527)	0.355
Heart failure	3497	212	6.06	3541	174	4.91	1.274 (1.043, 1.557)	0.018	1.234 (1.015, 1.499)	0.034
Cardiac arrhythmia	3283	381	11.61	3384	318	9.40	1.307 (1.126, 1.516)	<0.001	1.235 (1.073, 1.422)	0.003

**Males**										
All-cause mortality	2884	305	10.58	2903	245	8.44	1.200 (1.014, 1.419)	0.034	1.253 (1.068, 1.471)	0.006
Major adverse cardiovascular events	2656	282	10.62	2677	285	10.65	0.962 (0.816, 1.134)	0.647	0.997 (0.854, 1.165)	0.973
Acute myocardial infarction	2842	127	4.47	2859	132	4.62	0.927 (0.726, 1.183)	0.541	0.968 (0.763, 1.228)	0.788
Stroke	2879	93	3.23	2873	94	3.27	0.946 (0.710, 1.260)	0.704	0.987 (0.745, 1.309)	0.929
Heart failure	2745	198	7.21	2773	195	7.03	0.990 (0.812, 1.207)	0.922	1.026 (0.848, 1.241)	0.794
Cardiac arrhythmia	2545	325	12.77	2572	327	12.71	0.972 (0.834, 1.134)	0.720	1.004 (0.870, 1.159)	0.952
**Ages 18-59**										
All-cause mortality	2938	64	2.18	2954	55	1.86	1.122 (0.782, 1.609)	0.532	1.170 (0.819, 1.672)	0.388
Major adverse cardiovascular events	2899	78	2.69	2899	57	1.97	1.304 (0.927, 1.835)	0.127	1.368 (0.976, 1.918)	0.067
Acute myocardial infarction	2958	40	1.35	2953	22	0.75	1.708 (1.015, 2.874)	0.041	1.815 (1.082, 3.046)	0.022
Stroke	2957	26	0.88	2957	15	0.51	1.653 (0.875, 3.121)	0.117	1.733 (0.920, 3.266)	0.085
Heart failure	2930	45	1.54	2935	35	1.19	1.230 (0.791, 1.914)	0.357	1.288 (0.830, 1.997)	0.257
Cardiac arrhythmia	2806	152	5.42	2855	107	3.75	1.424 (1.112, 1.823)	0.005	1.445 (1.135, 1.841)	0.003
**Ages ≥60**										
All-cause mortality	3833	502	13.10	3820	431	11.28	1.122 (0.987, 1.277)	0.079	1.161 (1.029, 1.310)	0.015
Major adverse cardiovascular events	3432	509	14.83	3499	449	12.83	1.138 (1.002, 1.292)	0.046	1.156 (1.027, 1.300)	0.016
Acute myocardial infarction	3762	198	5.26	3794	146	3.85	1.338 (1.080, 1.657)	0.007	1.368 (1.110, 1.686)	0.003
Stroke	3794	176	4.64	3787	159	4.20	1.073 (0.865, 1.330)	0.522	1.105 (0.896, 1.363)	0.351
Heart failure	3572	381	10.66	3659	347	9.48	1.092 (0.944, 1.263)	0.235	1.125 (0.980, 1.291)	0.095
Cardiac arrhythmia	3264	591	18.11	3409	584	17.13	1.022 (0.911, 1.146)	0.712	1.057 (0.953, 1.173)	0.296

Supplementary table 6. **Detailed results of all cancer outcomes in dermatitis herpetiformis.**
Risk of solid cancers, hematological cancers, gastrointestinal cancers, non-Hodgkin lymphomas, breast cancer, and lung cancer in individuals with dermatitis herpetiformis. Enteropathy-associated T-cell lymphoma was included but returned null findings. P-values <0.0071 were considered statistically significant after Bonferroni correction for seven outcomes investigated. Statistics could not be calculated for breast cancer in the male and 18-59 subgroups due to insufficient number of events.
*Actual count is 1-10 since any count below 10 is aggregated due to data protection regulations.
CI: confidence interval; DH: dermatitis herpetiformis

Outcome	Exposed cohort (DH)			Unexposed cohort (comparators)			Hazard ratio (95% CI)	P-value	Risk ratio (95% CI)	P-value
	N total	N outcomes	Risk (%)	N total	N outcomes	Risk (%)				
**Unrestricted follow-up (primary analysis)**										
Solid cancers	6188	536	8.66	6310	485	7.69	1.123 (0.994, 1.270)	0.063	1.127 (1.002, 1.268)	0.046
Hematological cancers	6764	77	1.14	6831	59	0.86	1.303 (0.928, 1.829)	0.125	1.318 (0.941, 1.847)	0.108
Gastrointestinal cancers	6840	40	0.58	6839	46	0.67	0.854 (0.559, 1.305)	0.465	0.869 (0.570, 1.326)	0.516
Non-Hodgkin lymphomas	6822	44	0.64	6871	17	0.25	2.579 (1.473, 4.514)	0.001	2.607 (1.491, 4.558)	<0.001
Breast cancer	6774	81	1.20	6763	70	1.04	1.139 (0.827, 1.568)	0.426	1.155 (0.841, 1.588)	0.373
Lung cancer	6848	56	0.82	6850	55	0.80	1.000 (0.689, 1.451)	0.999	1.018 (0.703, 1.475)	0.923
**5-year follow-up (time-restricted analysis)**										
Solid cancers	6188	359	5.80	6310	347	5.50	1.055 (0.910, 1.223)	0.476	1.055 (0.914, 1.218)	0.464
Hematological cancers	6764	63	0.93	6831	39	0.57	1.632 (1.095, 2.433)	0.015	1.631 (1.096, 2.429)	0.015
Gastrointestinal cancers	6840	24	0.35	6839	35	0.51	0.684 (0.407, 1.150)	0.150	0.686 (0.408, 1.151)	0.151
Non-Hodgkin lymphomas	6822	36	0.53	6871	12	0.17	3.021 (1.572, 5.806)	<0.001	3.022 (1.573, 5.802)	<0.001
Breast cancer	6774	48	0.71	6763	54	0.80	0.884 (0.599, 1.304)	0.534	0.887 (0.602, 1.307)	0.545
Lung cancer	6848	36	0.53	6850	40	0.58	0.897 (0.572, 1.407)	0.635	0.900 (0.575, 1.411)	0.646
**Outcomes during the first six months excluded (sensitivity analysis S1)**										
Solid cancers	6118	466	7.62	6229	404	6.49	1.171 (1.025, 1.338)	0.020	1.174 (1.033, 1.335)	0.014
Hematological cancers	6739	52	0.77	6823	51	0.75	1.012 (0.688, 1.489)	0.952	1.032 (0.703, 1.517)	0.871
Gastrointestinal cancers	6838	38	0.56	6827	34	0.50	1.094 (0.688, 1.738)	0.705	1.116 (0.703, 1.770)	0.641
Non-Hodgkin lymphomas	6810	32	0.47	6868	14	0.20	2.265 (1.208, 4.246)	0.009	2.305 (1.231, 4.316)	0.007
Breast cancer	6770	77	1.14	6748	55	0.82	1.374 (0.972, 1.942)	0.071	1.395 (0.989, 1.969)	0.057

Lung cancer	6840	48	0.70	6841	46	0.67	1.021 (0.681, 1.530)	0.919	1.044 (0.697, 1.562)	0.835
**Implementing a required healthcare encounter ≥6 months before index (sensitivity analysis S2)**										
Solid cancers	4481	399	8.90	4616	397	8.60	1.049 (0.913, 1.206)	0.496	1.035 (0.907, 1.182)	0.608
Hematological cancers	5004	57	1.14	5068	49	0.97	1.192 (0.813, 1.746)	0.367	1.178 (0.806, 1.722)	0.397
Gastrointestinal cancers	5080	31	0.61	5084	35	0.69	0.893 (0.551, 1.449)	0.648	0.886 (0.547, 1.435)	0.624
Non-Hodgkin lymphomas	5055	32	0.63	5098	15	0.29	2.167 (1.174, 4.002)	0.011	2.151 (1.167, 3.968)	0.012
Breast cancer	5007	62	1.24	5021	61	1.21	1.036 (0.728, 1.476)	0.844	1.019 (0.717, 1.448)	0.915
Lung cancer	5082	44	0.87	5091	38	0.75	1.169 (0.758, 1.805)	0.480	1.160 (0.753, 1.787)	0.501
**Cohort definition requiring ≥2 codes of dermatitis herpetiformis (sensitivity analysis S3)**										
Solid cancers	2314	252	10.89	2362	187	7.92	1.130 (0.935, 1.365)	0.207	1.376 (1.149, 1.647)	0.000
Hematological cancers	2528	38	1.50	2545	20	0.79	1.601 (0.931, 2.753)	0.086	1.913 (1.116, 3.278)	0.016
Gastrointestinal cancers	2541	17	0.67	2534	13	0.51	1.051 (0.510, 2.168)	0.892	1.304 (0.635, 2.679)	0.469
Non-Hodgkin lymphomas	2541	24	0.94	2555	10*	0.39	2.246 (1.043, 4.834)	0.034	2.413 (1.156, 5.036)	0.015
Breast cancer	2529	26	1.03	2533	27	1.07	0.792 (0.462, 1.358)	0.395	0.964 (0.564, 1.648)	0.895
Lung cancer	2545	20	0.79	2549	21	0.82	0.755 (0.409, 1.396)	0.369	0.954 (0.518, 1.755)	0.879
**Cohort of dermatitis herpetiformis and dapsone treatment (sensitivity analysis S4)**										
Solid cancers	1563	169	10.81	1606	126	7.85	1.210 (0.960, 1.524)	0.106	1.378 (1.106, 1.717)	0.004
Hematological cancers	1727	26	1.51	1748	15	0.86	1.553 (0.822, 2.934)	0.171	1.754 (0.933, 3.301)	0.077
Gastrointestinal cancers	1745	13	0.74	1738	11	0.63	1.001 (0.448, 2.237)	0.998	1.177 (0.529, 2.620)	0.689
Non-Hodgkin lymphomas	1740	17	0.98	1755	10*	0.57	1.670 (0.744, 3.750)	0.209	1.715 (0.787, 3.734)	0.169
Breast cancer	1735	17	0.98	1736	19	1.09	0.790 (0.410, 1.520)	0.478	0.895 (0.467, 1.717)	0.739
Lung cancer	1750	15	0.86	1752	15	0.86	0.864 (0.422, 1.769)	0.688	1.001 (0.491, 2.042)	0.997
**Females**										
Solid cancers	3333	237	7.11	3419	244	7.14	1.013 (0.847, 1.211)	0.891	0.996 (0.839, 1.184)	0.967
Hematological cancers	3620	26	0.72	3646	25	0.69	1.057 (0.610, 1.831)	0.843	1.047 (0.606, 1.810)	0.868
Gastrointestinal cancers	3647	17	0.47	3658	23	0.63	0.764 (0.408, 1.430)	0.398	0.741 (0.397, 1.385)	0.346
Non-Hodgkin lymphomas	3642	15	0.41	3663	10*	0.27	1.549 (0.696, 3.447)	0.280	1.509 (0.679, 3.354)	0.310

Breast cancer	3560	77	2.16	3569	68	1.91	1.162 (0.838, 1.610)	0.368	1.135 (0.822, 1.568)	0.441
Lung cancer	3652	27	0.74	3664	20	0.55	1.379 (0.773, 2.459)	0.274	1.354 (0.761, 2.410)	0.300
**Males**										
Solid cancers	2611	270	10.34	2672	251	9.39	1.065 (0.897, 1.264)	0.475	1.101 (0.935, 1.296)	0.248
Hematological cancers	2866	49	1.71	2904	36	1.24	1.354 (0.881, 2.082)	0.166	1.379 (0.900, 2.114)	0.138
Gastrointestinal cancers	2913	21	0.72	2911	18	0.62	1.119 (0.596, 2.100)	0.727	1.166 (0.623, 2.184)	0.631
Non-Hodgkin lymphomas	2900	27	0.93	2925	17	0.58	1.562 (0.851, 2.866)	0.147	1.602 (0.875, 2.933)	0.123
Breast cancer	2941	10*	0.34	2942	10*	0.34	-	-	-	-
Lung cancer	2917	28	0.96	2927	22	0.75	1.235 (0.706, 2.159)	0.459	1.277 (0.732, 2.227)	0.387
**Ages 18-59**										
Solid cancers	2611	270	10.34	2672	251	9.39	1.065 (0.897, 1.264)	0.475	1.101 (0.935, 1.296)	0.248
Hematological cancers	2866	49	1.71	2904	36	1.24	1.354 (0.881, 2.082)	0.166	1.379 (0.900, 2.114)	0.138
Gastrointestinal cancers	2913	21	0.72	2911	18	0.62	1.119 (0.596, 2.100)	0.727	1.166 (0.623, 2.184)	0.631
Non-Hodgkin lymphomas	2900	27	0.93	2925	17	0.58	1.562 (0.851, 2.866)	0.147	1.602 (0.875, 2.933)	0.123
Breast cancer	2941	10*	0.34	2942	10*	0.34	-	-	-	-
Lung cancer	2917	28	0.96	2927	22	0.75	1.235 (0.706, 2.159)	0.459	1.277 (0.732, 2.227)	0.387
**Ages ≥60**										
Solid cancers	3316	458	13.81	3415	471	13.79	0.983 (0.864, 1.118)	0.788	1.001 (0.889, 1.128)	0.981
Hematological cancers	3809	65	1.71	3837	47	1.22	1.371 (0.942, 1.997)	0.098	1.393 (0.960, 2.022)	0.080
Gastrointestinal cancers	3869	33	0.85	3875	43	1.11	0.754 (0.479, 1.188)	0.222	0.769 (0.489, 1.207)	0.252
Non-Hodgkin lymphomas	3856	37	0.96	3880	22	0.57	1.663 (0.981, 2.821)	0.056	1.692 (1.000, 2.863)	0.047
Breast cancer	3814	60	1.57	3819	55	1.44	1.064 (0.737, 1.534)	0.742	1.092 (0.760, 1.571)	0.633
Lung cancer	3871	48	1.24	3889	52	1.34	0.887 (0.599, 1.315)	0.550	0.927 (0.628, 1.369)	0.704

Supplementary table 7. **Detailed results of additional sensitivity analyses.**
Propensity-score matching including race/ethnicity includes analyses for all outcomes and an analysis of all-cause mortality in cohort definitions excluding prior inflammatory bowel disease and gastrointestinal cancers.
*Actual count is 1-10 since any count below 10 is aggregated due to data protection regulations.
CI: confidence interval

Outcome	Exposed cohort			Unexposed cohort (comparators)			Hazard ratio (95% CI)	P-value	Risk ratio (95% CI)	P-value
	N total	N outcomes	Risk (%)	N total	N outcomes	Risk (%)				
**Inclusion of race/ethnicity in the propensity-score matching, results for coeliac disease**										
Mortality	216,218	9,156	4.23	216,664	8,505	3.93	1.127 (1.094–1.161)	<0.001	1.079 (1.048–1.110)	<0.001
MACE	210,817	10,386	4.93	211,655	9,837	4.65	1.121 (1.090–1.152)	<0.001	1.060 (1.032–1.089)	<0.001
Acute myocardial infarction	217,220	3,449	1.59	217,517	3,381	1.55	1.079 (1.029–1.132)	0.002	1.022 (0.975–1.071)	0.375
Stroke	216,938	3,720	1.71	217,162	3,345	1.54	1.174 (1.121–1.231)	<0.001	1.113 (1.063–1.166)	<0.001
Heart failure	214,540	7,044	3.28	214,936	6,745	3.14	1.104 (1.068–1.141)	<0.001	1.046 (1.012–1.081)	0.007
Cardiac arrhythmia	202,273	17,301	8.55	207,030	15,687	7.58	1.198 (1.172–1.224)	<0.001	1.129 (1.106–1.152)	<0.001
Solid cancers	206,525	10,477	5.07	208,236	11,148	5.35	0.994 (0.968–1.021)	0.662	0.948 (0.923–0.973)	<0.001
Hematological cancers	217,602	1,694	0.78	218,371	1,232	0.56	1.451 (1.348–1.561)	<0.001	1.380 (1.283–1.485)	<0.001
GI cancers	218,566	1,029	0.47	218,834	986	0.45	1.094 (1.002–1.194)	0.044	1.045 (0.958–1.140)	0.323
Non-Hodgkin lymphomas	218,747	809	0.37	219,155	524	0.24	1.624 (1.455–1.813)	<0.001	1.547 (1.386–1.726)	<0.001
Breast cancer	216,781	2,040	0.94	216,746	2,367	1.09	0.905 (0.853–0.960)	0.001	0.862 (0.812–0.914)	<0.001
Lung cancer	219,180	885	0.40	219,115	1,071	0.49	0.867 (0.793–0.948)	0.002	0.826 (0.756–0.903)	<0.001
EATL	219,733	24	0.01	219,750	10*	0.00	12.573 (2.971–53.212)	<0.001	2.400 (1.148–5.019)	0.016
**Inclusion of race/ethnicity in the propensity-score matching, results for dermatitis herpetiformis**										
Mortality	7,481	607	8.11	7,499	495	6.60	1.194 (1.060–1.344)	0.003	1.229 (1.096–1.378)	<0.001
MACE	6,987	628	8.99	7,146	595	8.33	1.047 (0.936–1.172)	0.419	1.079 (0.970–1.202)	0.162
Acute myocardial infarction	7,408	243	3.28	7,486	209	2.79	1.140 (0.948–1.372)	0.164	1.175 (0.979–1.410)	0.082
Stroke	7,447	214	2.87	7,453	196	2.63	1.059 (0.872–1.285)	0.563	1.093 (0.903–1.323)	0.363
Heart failure	7,175	458	6.38	7,326	431	5.88	1.051 (0.922–1.199)	0.457	1.085 (0.955–1.232)	0.209
Cardiac arrhythmia	6,713	810	12.07	6,917	744	10.76	1.097 (0.993–1.212)	0.068	1.122 (1.021–1.232)	0.016
Solid cancers	6,838	584	8.54	6,991	519	7.42	1.132 (1.005–1.274)	0.040	1.150 (1.027–1.289)	0.015
Hematological cancers	7,462	81	1.09	7,548	71	0.94	1.128 (0.820–1.551)	0.460	1.154 (0.840–1.584)	0.375

GI cancers	7,551	46	0.61	7,550	58	0.77	0.773 (0.525–1.138)	0.190	0.793 (0.539–1.166)	0.237
Non-Hodgkin lymphomas	7,526	49	0.65	7,586	29	0.38	1.665 (1.052–2.635)	0.028	1.703 (1.077–2.693)	0.021
Breast cancer	7,474	83	1.11	7,486	68	0.91	1.189 (0.863–1.638)	0.290	1.223 (0.889–1.682)	0.216
Lung cancer	7,556	57	0.75	7,575	59	0.78	0.944 (0.656–1.358)	0.755	0.969 (0.674–1.392)	0.863
**Mortality in patients with coeliac disease after exclusion of prior inflammatory bowel disease and gastrointestinal cancers**										
All-cause mortality	206,499	8,365	4.05	207,217	7,752	3.74	1.114 (1.080–1.149)	<0.001	1.083 (1.051–1.116)	<0.001
**Mortality in patients with dermatitis herpetiformis after exclusion of prior inflammatory bowel disease and gastrointestinal cancers**										
All-cause mortality	7,141	559	7.83	7,157	411	5.74	1.271 (1.119–1.444)	<0.001	1.363 (1.205–1.542)	<0.001
